# Targeting the gut microbiome for type 2 diabetes management: a scoping review of systematic reviews and meta-analyses

**DOI:** 10.3389/fendo.2026.1682174

**Published:** 2026-01-29

**Authors:** Rongsheng Jiang, Likun Zheng, Jinxu Fang, Qifan Guan, Hao Yuan, Jianfeng Liang, Jingwen Zhang, Qingxuan Han, Mingjun Liu

**Affiliations:** 1 College of Acupuncture and Tuina, Changchun University of Chinese Medicine, Changchun, Jilin, China; 2 Department of Health and Welfare, Changchun Humanities and Sciences College, Changchun, Jilin, China

**Keywords:** current status, gut microbiome, research quality, scope review, type 2 diabetes mellitus

## Abstract

**Background:**

The Gut Microbiome (GM) is now a novel target for the treatment of Type 2 Diabetes Mellitus (T2DM), and several systematic reviews and Meta-analyses have provided evidence on the efficacy and safety of modulating GM in T2DM, but this evidence has not been consolidated.

**Objective:**

The purpose of this scoping review was to summarize the currently available evidence and to assess the breadth and quality of these systematic reviews and Meta-analyses.

**Methods:**

This study was guided by the PRISMA Extension for Scoping Reviews (PRISMA ScR) and the Arksey and O’Malley methodological framework. Electronic searches were conducted in multiple databases from the time of construction to May 1, 2025. Systematic reviews and Meta-analyses of regulatory GM to improve T2DM were included. 2 researchers independently screened full text, extracted review characteristics, and assessed methodological quality using the AMSTAR2 scale tool.

**Result:**

A total of 23 systematic reviews and Meta-analyses were included, which were published in 2015-2024.Probiotics/synbiotics were the most commonly used interventions; the included studies were generally of low methodological quality (only 1 was of high quality); most of the studies reported an improvement in some glycemic and lipid markers by modulating the microbiota, but there was heterogeneity in the results; and there was insufficient attention to adverse events.

**Conclusion:**

The available evidence suggests that regulating GM may be beneficial, but is limited by the quality of the studies, and future studies with large samples, long-term follow-up, and standardized adverse event reporting are needed to demonstrate its safety and long-term effectiveness conclusively.

**Systematic Review Registration:**

https://doi.org/10.17605/OSF.IO/PW28U.

## 1 Introduction

T2DM is a globally prevalent metabolic disease (1), and it is expected that the number of people with T2DM will reach 700 million by 2045 (2). It has become a significant challenge for the global health system. The main manifestations of T2DM are insufficient insulin secretion and insulin resistance (IR), and its development is strongly related to the genetic and external environments, such as diet, exercise, and lifestyle habits (3, 4). Despite the large amount of research conducted by scholars, the pathogenesis of T2DM has not yet been clarified. In addition to the abnormalities of glucose metabolism and lipid metabolism (5), there are many complications of T2DM, such as cardiovascular complications, cognitive impairment, retinopathy, and renal injury (6–8), which seriously affects the quality of life of the patients and brings a heavy medical and economic burden. Because the pathogenesis of T2DM is complex and has many complications, it faces diverse challenges in clinical treatment. Currently, treatment for T2DM remains predominantly pharmacological, primarily encompassing biguanides, sulphonylureas, thiazolidinediones, DPP-4 inhibitors, SGLT-2 inhibitors, GLP-1 receptor agonists, insulin, and alpha-glucosidase inhibitors (9, 10). Although good results have been achieved in the control of blood glucose, the economic pressure of long-term medication and the side effects that cannot be ignored still make patients with T2DM face unsatisfactory treatment. Based on this status quo, it is necessary to explore new therapeutic targets and develop new treatment strategies to cope with the shortcomings of current mainstream regimens (11).

The human gut is a complex ecosystem containing about 100 trillion commensal microorganisms, with the phylum Thick-walled Bacteria and the phylum Mycobacterium being the main components (12). These microorganisms not only prevent pathogenic infections, facilitate the digestion and absorption of nutrients, and synthesize essential vitamins and amino acids, but also exert anti-inflammatory effects, regulate lipid metabolism, and positively regulate T2DM through the production of metabolites, such as short-chain fatty acids (SCFAs), lipopolysaccharide (LPS), and trimethylamine N-oxide (13–15). With the continuous development of technology, an extensive cohort study using macro-genomics found a link between T2DM and GM (16), and a Mendelian randomization study further confirmed the causal relationship (17–20). In subsequent studies, scholars have intervened through drugs, diet, and fecal microbiota transplantation (FMT) and have found that these intervention modalities have a modulating effect on GM composition and have shown beneficial effects in individuals with T2DM (21).Although FMT has demonstrated potential efficacy, it is important to note that this therapy carries a risk of adverse reactions (22, 23). Further research should be conducted to refine this approach and enhance its safety profile. Meanwhile, SCFAs, which constitute a significant metabolite of GM, were found to inhibit the inflammatory response and promote the production of glucagon-like peptide-1 (GLP-1) in individuals with T2DM, thereby increasing insulin sensitivity (24). Bile acids, on the other hand, have been found to reduce gluconeogenesis, increase energy expenditure, and bind to the farnesol X receptor to enhance glucose uptake via ectopic glucose transporter protein 4 (25). Therefore, microbiota is considered a novel target for the treatment of T2DM (26).

GM changes in response to physiological and pathological changes in the host and can inversely regulate the state of the organism. Studies have shown significant differences in the gut microbiota of T2DM patients compared to non-diabetic individuals, in particular, an increase in pro-inflammatory bacteria (such as Escherichia coli, the family Vibrionaceae, and Clostridium difficile) and a decrease in anti-inflammatory bacteria (such as Clostridium prausimmus, Roseburia, Faecalibacterium, Akkermansia, Bifidobacterium, etc.) (27). In animal models, the association between GM and T2DM has likewise been demonstrated. Compared to normal Wistar rats, the GM of Goto-Kakizaki (GK) rats—a hereditary type 2 diabetes animal model—exhibited significant enrichment of genera including Prevotella_9, Blautia, Roseburia, Allobaculum, and Prevotella_1 (28).Similarly, db/db mice exhibit abnormal gut microbiota composition, and FMT induces weight gain, elevated fasting blood glucose levels, and altered microbiota composition in pseudo-germ-free mice (29).GM also plays a role in complications associated with T2DM. Previous studies have found that the genera Escherichia-Shigella and Prevotella_9 can accurately distinguish whether T2DM patients have nephropathy, while Prevotella_9 can accurately differentiate T2DM patients without nephropathy from healthy controls (30). In T2DM patients with cognitive impairment, alterations in gut microbiota composition were similarly observed, characterized by reduced abundances of Bifidobacteria, Tenericutes and unclassified RF39, alongside increased abundances of Peptostreptococcus and unclassified Leucotrichales (31). This appears to offer novel insights for diagnosing cognitive impairment in T2DM. Age, gender, population group and dietary habits affect GM composition and abundance and influence GM metabolites and host phenotype (32, 33). Evidence indicates that GM responds to changes in female sex hormone status and exacerbates metabolic dysfunction (34). Concurrently, scholars have identified differences in hypothalamic-pituitary-gonadal axis hormone signaling driven by the gut microbiota between male and female recipients (35). Exogenous nutritional and pharmacological interventions have significant effects on the organismal GM (36). Probiotic supplementation can effectively improve the microbiota structure of individuals with T2DM and alleviate the characteristic manifestations such as inflammation and IR (37–39). FMT has been used for thousands of years in the treatment of gastrointestinal diseases. This method has also been applied in the study of T2DM, and some new findings have been obtained. Sterile db/db mice receiving FMT from healthy mice showed significant reductions in Desulfovibrio and Clostridium coccoides levels and increased Akkermansia levels in the gut, along with improved blood glucose levels (40). In clinical studies, FMT also appears to show potential to enhance T2DM, with one study showing improvements in HbA1c and glycemic parameters in patients with T2DM after receiving a colony transplant from a healthy individual (41). Dendrobiumloddigesii, Gegen Qinlian Decoction have similarly shown potential to modulate the microbiota of individuals with T2DM and improve glycemic parameters, with herbal extracts as well as herbal formulations showing promising effects (42–44).

Although numerous studies have demonstrated the positive effects of modulating GM on glucose metabolism disorders in T2DM, researchers have conducted mechanistic studies on this topic. However, not all studies have been adequate, and the same unsuccessful phenomenon exists in studies where probiotics and FMT have intervened (45, 46). Therefore, in the face of this controversial phenomenon, we conducted this scoping review to summarize the evidence from current systematic reviews and Meta-analyses of the regulation of GM to improve T2DM and further assess the quality of this evidence. This will help to identify the current status and gaps in current research and provide a valuable reference for subsequent study design.

## 2 Materials and methods

This scoping review was conducted by the PRISMA ScR (47). The proposed scoping review is based on the methodological framework of Arksey and O’Malley (48). The methodology consists of six stages: (1) identification of the research question; (2) identification of relevant studies; (3) study selection; (4) graphing of the data; (5) collating, summarizing, and reporting the findings; and (6) consultation with key stakeholders (optional). The program for the scoping review is registered on the Open Science Framework platform (https://doi.org/10.17605/OSF.IO/PW28U).

To conduct a more comprehensive analysis of existing research, we summarized the included articles using bibliometric analysis methods. Specifically, we employed an online tool based on RStudio for the analysis (https://www.bibliometrix.org/home/).

### 2.1 Define the research question

The key aims of this review are as follows:

1. To depict the current status of regulating GM to improve T2DM.
2. To sort out the methods and application scope of regulating GM to improve T2DM.
3. Investigate the effects of regulating GM on T2DM.
4. To explore whether there are gaps in current research, as well as to identify future research directions.

### 2.2 Identify relevant research

#### 2.2.1 Search strategy

This review was searched exclusively through electronic databases; the databases searched included PubMed, Web of Science, Cochrane Library, and Embase. The searches were carried out using MeSH words and free words. The searches were carried out using MeSH words and free words. The main search terms included “Diabetes Mellitus, Type 2, “Gastrointestinal Microbiome, “Systematic review,” “meta-analysis,” and their synonyms. **Table 1** shows the PubMed search strategy. The search was conducted from database construction to May 1, 2025.

**Table 1:** Search strategy (Pubmed as an example).

Rank	Search strategy	Results
#1	Diabetes Mellitus, Type 2[MeSH]	190021
#2	Diabetes Mellitus, Type 2 OR Diabetes Mellitus, Non Insulin Dependent OR Stable Diabetes Mellitus OR Diabetes Mellitus, Type II OR NIDDM OR Diabetes Mellitus, Noninsulin Dependent OR Type 2 Diabetes Mellitus OR Noninsulin Dependent Diabetes Mellitus OR Type 2 Diabetes OR Diabetes, Type 2 OR T2DM	288151
#3	#1 OR #2	288293
#4	Gastrointestinal Microbiome[MeSH]	55437
#5	Gastrointestinal Microbiome* OR Microbiome, Gastrointestinal OR Gut Microbiome* OR Microbiome, Gut OR Gut Microflora OR Microflora, Gut OR Gut Microbiota* OR Microbiota, Gut OR Gastrointestinal Flora OR Flora, Gastrointestinal OR Gut Flora OR Flora, Gut OR Gastrointestinal Microbiota* OR Microbiota, Gastrointestinal OR Gastrointestinal Microbial Community* OR Gastrointestinal Microflora OR Microflora, Gastrointestinal OR Gastric Microbiome* OR Microbiome, Gastric OR Intestinal Microbiome* OR Microbiome, Intestinal OR Intestinal Microbiota* OR Microbiota, Intestinal OR Intestinal Microflora OR Microflora, Intestinal OR Intestinal Flora OR Flora, Intestinal	132285
#6	#4 OR #5	132317
#7	Systematic review OR meta-analysis	526426
#8	#3 and #6 and #7	154

#### 2.2.2 Inclusion/exclusion criteria

Inclusion Criteria: ① Type of study: published studies related to the modulation of GM to improve T2DM, with no restriction on language and region; ② Subjects: patients with a diagnosis of T2DM; ③ Interventions: RCTs with regulation of GM as the primary intervention were included; SR/MA should report or assess baseline comparability of included RCTs. ④ Outcome Indicators: changes in GM and T2DM-related indicators; ⑤ Publication type: systematic review and Meta-analysis.

Exclusion criteria: (1) duplicate publications (keep the latest version); (2) publications that were not of the type of systematic reviews and Meta-analyses; (3) publications with incomplete data or unavailable full text; and (4) publications related to animal experiments.

### 2.3 Screening of publications

2 researchers carried out publication screening. Publications were first imported into the literature management software EndNote X9, duplicates were eliminated, and the remaining records were assessed. During the screening process, two researchers (Likun Zheng and Jinxu Fang) assessed any potentially eligible publications. They recorded the reasons for exclusion, and when disagreements arose, they were discussed and agreed upon with a third researcher (Hao Yuan).

### 2.4 Charting the data

We developed a standardized data extraction protocol based on the research questions to extract key data from the included studies. Data extraction was done by Zhengri Cong and Jianfeng Liang and checked by another researcher, Jingwen Zhang.

The extracted variables were categorized as follows: 1. basic information: first author, year of publication, journal of publication, and type of grant; 2. participant characteristics: patient age, sample size; 3. methodological details: study design, type of intervention, type of comparator, and outcome metrics; and 4. results and discussion: significant findings, conclusions, limitations, and recommendations for future research.

### 2.5 Collating, summarizing, and reporting findings

Analysis: both quantitative analysis based on numerical statistics (i.e., characteristics of included studies, analysis of main results) and qualitative analysis through narrative synthesis. Quantitative analysis used data from the included studies, with categorical variables represented using numbers and percentages, and continuous variables represented using tables or graphs. In qualitative analysis, the focus is on a discussion of the available evidence as a way of determining whether there are gaps in the current research and identifying directions for future research.

Reporting: data were extracted from the included studies and analyzed. Findings were briefly organized in tabular form and analyzed using narrative descriptions. As a scoping study, no subgroup analysis or sensitivity analysis was performed on the data.

### 2.6 Quality assessment

2 researchers (Qifan Guan and Likun Zheng) assessed the quality of the included studies using the AMSTAR2 instrument in duplicate. Any disagreements were resolved by a third researcher (Qingxuan Han). The AMSTAR2 scale consists of 16 items (49): each item can be answered with either “yes” or “no,” some items can also be answered with Each item can be answered with “yes” or “no,” and some items can be answered with “partially yes.” The methodological quality of the study was considered high if all items were free of flaws or if only one non-critical item was flawed. Methodological quality was judged to be moderate when more than one non-critical item was defective, but no critical items were defective. Method quality was low when one essential item was defective, with or without non-critical item defects. When more than one critical item is defective and there are or are not defects in non-critical items, the method quality is very low.

## 3 Results

### 3.1 Overview of included articles

**Figure 1:**
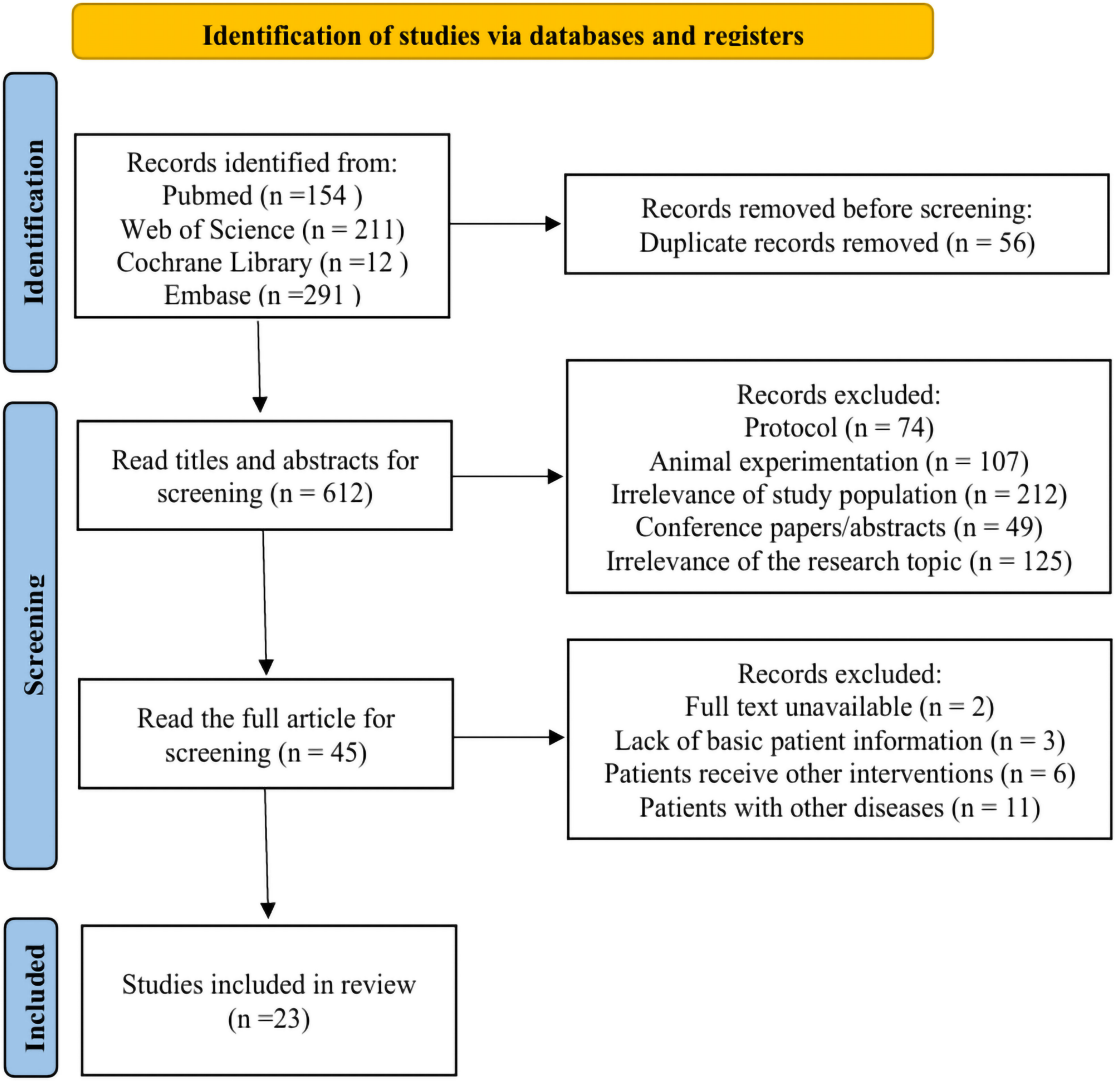
Publication screening process.

A total of 668 publications (Pubmed 154, Web of Science 211, Cochrane Library 12 Embase 291) were retrieved, with 612 remaining after excluding duplicates, 45 remaining after reading the titles and abstracts, and excluding non-human trials and publications not related to the study topic, further reading of the full text excluded publications where the intervention method and target population did not meet the inclusion criteria, resulting in a final selection of 23 eligible articles. 23 articles (50–72) were published between 2015 and 2024; 22 performed a meta-analysis to determine outcomes, and 1 performed a systematic review only. The number of authors per study ranged from 2 to 8, with a mean number of authors of 5.78 ± 1.98. The number of RCTs included in each study ranged from 8 to 37, with a median of 12. Following collation of the original studies included in the 23 articles, a total of 142 distinct original studies were identified. The size of participants in each study ranged from 221 to 2502, with a median of 714 subjects. **Figure 1** illustrates the detailed process of publication screening, and **Supplementary Table 1** shows the details of the included studies.

**Figure 2:**
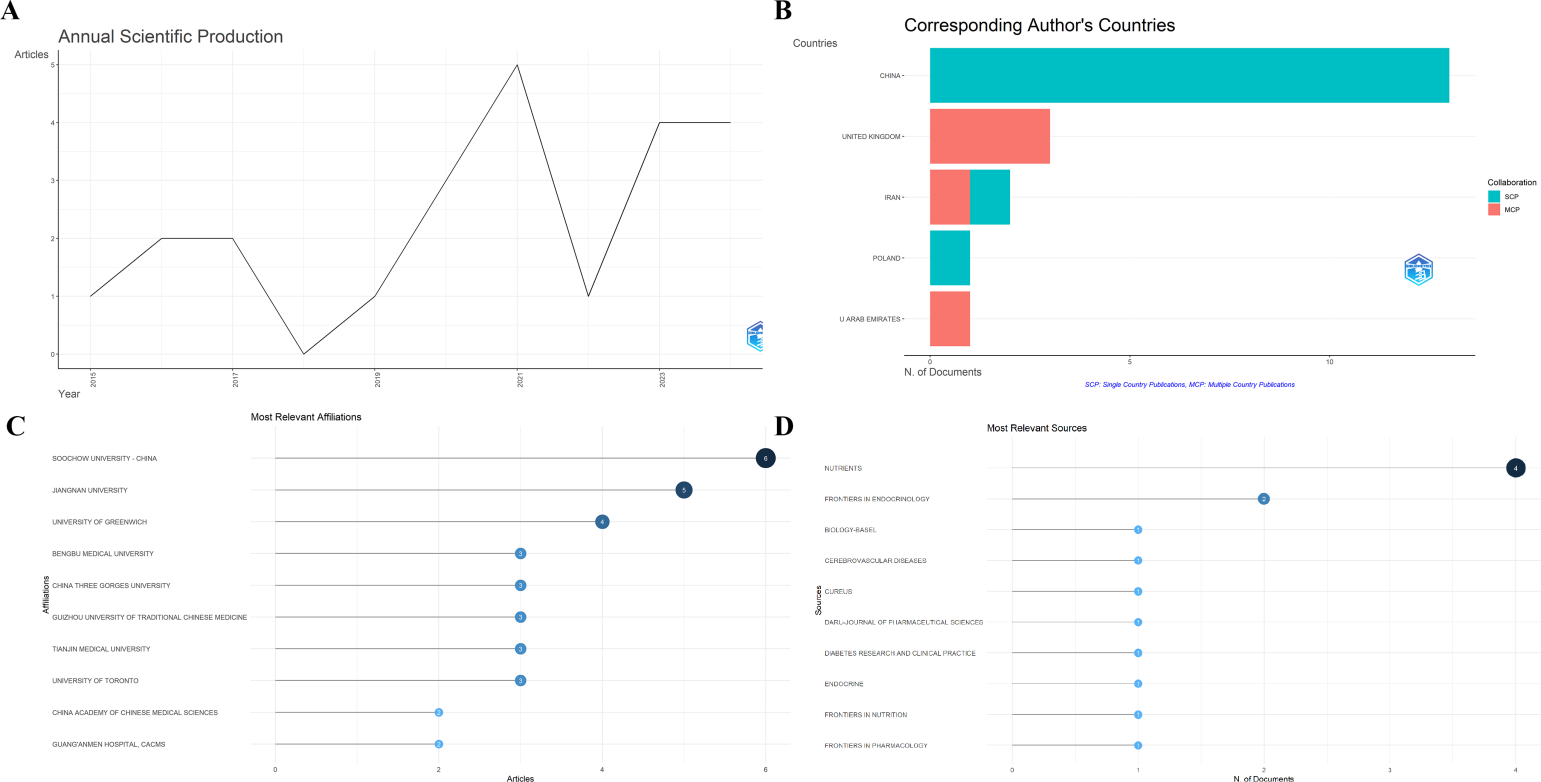
**(A)** Annual publication volume **(B)** Most relevant countries **(C)** Most relevant institutions **(D)** Most relevant journals.

**Figure 3:**
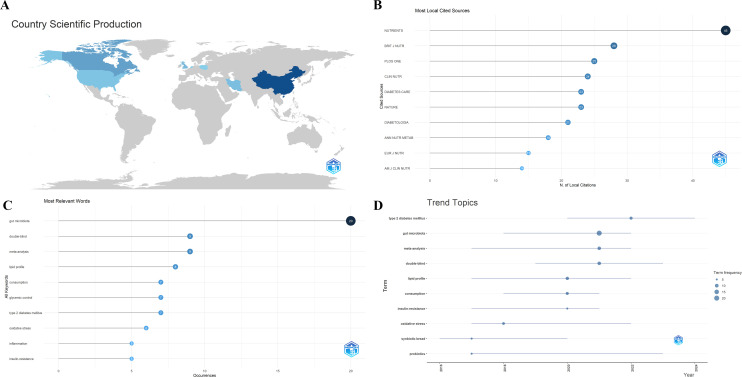
**(A)** Country Science Production **(B)** Most local cited sources **(C)** Most relevant words **(D)** Trend topics.

Bibliometric analysis reveals that the highest number of publications occurred in 2021, reaching five articles, while 2018 recorded the lowest output with zero publications (**Figure 2A**). By corresponding author affiliation, China contributed the most publications, followed by the United Kingdom and Iran (**Figure 2B**). The most prolific institutions were Soochow University, Jiangnan University, and the University of Greenwich (**Figure 2C**). The journals with the highest publication counts were Nutrients and Frontiers in Endocrinology, followed by Biology-Basel (**Figure 2D**). **Figure 3A** illustrates national scientific productivity, with China ranking first in frequency (frequency: 43), followed by Canada (frequency: 15) and the United Kingdom (frequency: 8). **Figure 3B** presents the most frequently cited journals, with Nutrients (cited 45 times) ranking first, followed by the British Journal of Nutrition (cited 28 times) and PLOS ONE (cited 25 times); **Figure 3C** displays high-frequency keywords, with gut microbiota (frequency: 20) appearing most frequently, followed by double-blind (frequency: 9) and meta-analysis (frequency: 9); **Figure 3D** displays trending topics, with the longest-running theme being probiotics (2017–2023), while recent themes include type 2 diabetes mellitus (2020–2024) and double-blind (2019–2023).

### 3.2 Search strategy of included studies

A total of 19 databases were searched. The range of databases used per study was 1-7, the median number of databases used was 4, and the mean was 4 ± 1.51; the most commonly used databases were PubMed (19/23), Cochrane Library (15/23), Embase (15/23), and Web of Science (14/23). 22 studies were searched starting from database building, and 1 study (56) searched a specific time frame (2017-2023). 21 studies searched only electronic databases, 1 study also searched references of the included studies, and 1 study also searched the study registration platform.

### 3.3 Inclusion/exclusion criteria for included studies

All studies had no restrictions such as gender and ethnicity, 7 studies included patients ≥18 years of age, 1 study included patients 20–80 years of age, 2 studies included adults, and 13 studies had no restrictions on age. The intervention modality for all RCTs included in the studies was adjustable GM and placebo or conventional intervention was used in the control group. The outcome indicators were all directly related to T2DM, such as glycolipid metabolism, inflammatory markers, and GM changes.

### 3.4 Intervention methods used in the included studies

**Table 2** shows the intervention methods used in the original research. The 23 included studies had a total of 338 intervention groups and 338 control groups. There were 6 main categories of intervention methods used, with Probiotics/Hybrids (n=214, 63.3%) being the most commonly used category of intervention, followed by Prebiotics/Dietary Fiber (n=62, 18.3%) and Dietary Pattern Modification (n=31, 9.2%). There were 3 main categories of interventions used by the control group, with Placebo (n=254, 75.1%) and conventional diet/medicine (n=64, 18.9%) being the most commonly used interventions, and active control (n=20, 5.9%) being the least represented intervention.

**Table 2:** Intervention modalities for SR/ME included.

Groups	Types of intervention (frequency)	Main forms
Test Group	Probiotics/synbiotics(214)	Capsules Yogurt Powder Fermented Milk Tablets Bread Soy Milk
	Prebiotics/dietary fiber(62)	Inulin Resistant dextrin Resistant starch β-glucan Guar gum Plantago ovata
	Dietary pattern modification(31)	High Fiber Diet Fibre-Rich Macrobiotic Ma-Pi 2 Diet Mediterranean Diet Ketogenic Diet Almond Low Carb Diet
	Metformin + probiotics (18)	Capsules: metformin + probiotic complex capsules Yogurt: metformin + probiotic-fortified yogurt
	Compound Chinese medicines + Metformin (12)	Gegen Qinlian Decoction Shenlin Baizhu Powder Jinmai Wendan Decoction+Oads Maren Wan Qingfei Xiegan Decoction Tonifying Qi And Strengthening Spleen Decoction Qingre Huoxue Huatan Formula Wenyang Yiqi Huoxue Formula
	Fecal microbial transplantation (1)	FMT
Control group	Placebo (254)	Maltodextrin Starch capsules Microcrystalline cellulose Glycols Saline
	Regular diet/medication (64)	Regular yogurt/milk Metformin alone Standard diabetic diet Control Bread
	Active control (20)	Maltodextrin vs. resistant dextrin Whole wheat flour vs. arabinoxylan fiber Regular soy milk vs. probiotic soy milk

Probiotic strains were listed in 214 trial groups. *Lactobacillus acidophilus* was the most frequent strain (n=172, 80.4%), followed by *Bifidobacterium lactis* (n=158, 73.8%) and *Lactobacillus casei* (n=149, 69.6%). Most probiotic groups used composite strain combinations (n=203, 94.9%), with the most common strain combinations being *L. acidophilus* + *B. lactis* + *S. thermophilus* (n=48) and *L. acidophilus* + *L. casei* + *B. bifidum* + *B. lactis* + *L. rhamnosus* + *S. thermophilus* (n=32), *L.* sp*orogenes* + *B. coagulans* (n=26).

A total of 337 trials provided a specific intervention period, with intervention periods mostly clustered between 12 and 16 weeks (n=126, 37.4%). The shortest intervention period was 4 days, and the longest was 2 years; the mean intervention period was 8.21 weeks. The most frequent occurrence was an intervention period of 12 weeks (n=98, 29.1%).

### 3.5 Evaluation of the content of the included studies

**Figure 4:**
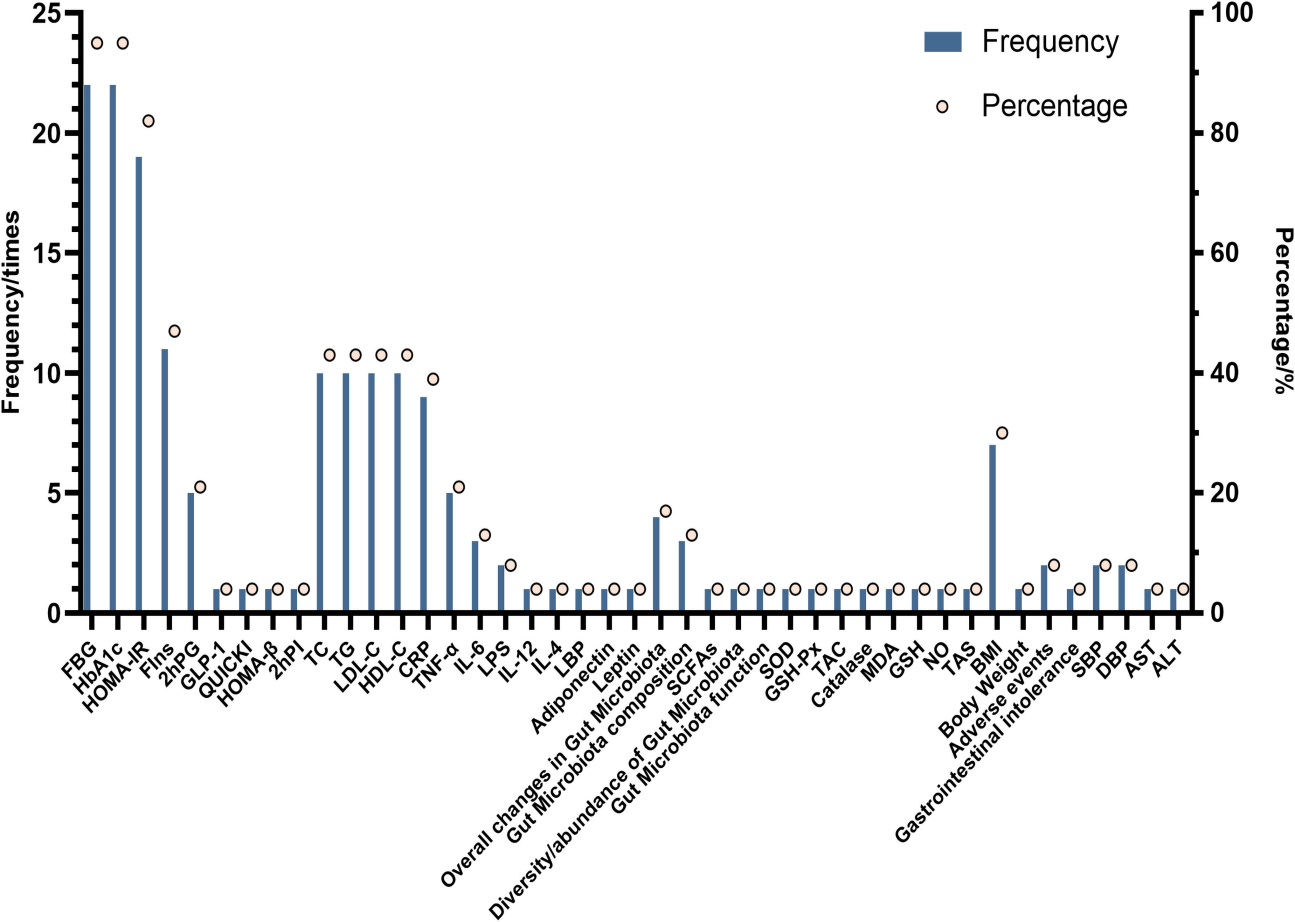
Evaluation elements of publications included.

23 studies’ outcome indicators mainly included Glycemic Control, Lipid Profile, Inflammatory Markers, Gut Microbiota, Oxidative Stress Markers, Anthropometric Measures, Adverse Events, Blood Pressure, and Liver Function.FBG and HbA1c were the most frequently occurring indicators (n=22, 95.65%), followed by HOMA-IR (n=19, 82.61%) and Fasting Insulin (n=10. 43.48%); Lipid Profile indicators mainly included TC, TG, HDL-C, and LDL-C (n=10, 43.48%); and Inflammatory Markers mainly included CRP (n=9, 39.13%), TNF-α (n=6. 26.09%), IL-6 (n=4, 17.39%); Gut Microbiota Indicators mainly included Overall changes in Gut Microbiota (n=4, 17.39%), Gut Microbiota composition (n=3, 13.04%), and also SCFAs, Diversity/abundance of Gut Microbiota, and Gut Microbiota function. While Oxidative Stress Markers, Anthropometric Measures, Blood Pressure, and Liver Function appeared less frequently, the Adverse Events correlation metrics appeared only 3 times. **Figure 4** demonstrates the frequency and percentage of the primary outcome indicators. **Table 3** summarizes the details of the outcome indicators included in the study.

**Table 3:** Outcome indicators for SR/ME included.

Indicator category	Specific indicators	Frequency	Percentage
Glycemic Control	FBG	22	95.65%
	HbA1c	22	95.65%
	HOMA-IR	19	82.61%
	FIns	11	47.83%
	2hPG	5	21.74%
	GLP-1	1	4.35%
	QUICKI	1	4.35%
	HOMA-β	1	4.35%
	2hPI	1	4.35%
Lipid Profile	TC	10	43.48%
	TG	10	43.48%
	LDL-C	10	43.48%
	HDL-C	10	43.48%
Inflammatory Markers	CRP	9	39.13%
	TNF-α	5	21.74%
	IL-6	3	13.04%
	LPS	2	8.7%
	IL-12	1	4.35%
	IL-4	1	4.35%
	LBP	1	4.35%
	Adiponectin	1	4.35%
	Leptin	1	4.35%
Gut Microbiota	Overall changes in Gut Microbiota	4	17.39%
	Gut Microbiota composition	3	13.04%
	SCFAs	1	4.35%
	Diversity/abundance of Gut Microbiota	1	4.35%
	Gut Microbiota function	1	4.35%
Oxidative Stress Markers	SOD	1	4.35%
	GSH-Px	1	4.35%
	TAC	1	4.35%
	Catalase	1	4.35%
	MDA	1	4.35%
	GSH	1	4.35%
	NO	1	4.35%
	TAS	1	4.35%
Anthropometric Measures	BMI	7	30.43%
	Body Weight	1	4.35%
Adverse Events	Adverse events	2	8.7%
	Gastrointestinal intolerance	1	4.35%
Blood Pressure	SBP	2	8.7%
	DBP	2	8.7%
Liver Function	AST	1	4.35%
	ALT	1	4.35%

### 3.6 Quality of included studies

**Supplementary Table 2** shows the results of the quality assessment of the included studies using the AMSTAR2 scale.1 study was of high quality, 6 studies were of low quality, and 16 studies were of very low quality. All studies fulfilled items 1, 5, 6, 8, 9, 11-13, and 16. In item 2, 11 studies did not provide a systematic evaluation of the study protocol identified in advance; in item 3, 6 studies revealed the reasons for inclusion of the RCT study; in item 4, only 1 study searched for complementary scopes outside of the electronic databases (grey literature, trial registration platforms); and for items 7 and 10, only 1 study provided a list of relevant studies that were entered into full-text reading at the screening stage, and none of the studies provided the source of funding for the included studies; for item 14, 2 studies did not provide a satisfactory explanation or discussion of heterogeneity; and for item 15, 11 studies used a graphical or statistical test for publication bias and discussed the likelihood of publication bias and the impact on the results. For studies without Meta-analysis (51), AMSTAR 2 items 11–15 are marked as Not Applicable.

## 4 Discussion

This is the first scoping review of systematic reviews and Meta-analyses of regulating GM to improve T2DM as far as we are aware. We conducted the study according to the PRISMA ScR and ‘Arksey and O’Malley’s methodological framework, integrating current evidence through a comprehensive search and rigorous screening, and critically assessing the included studies using the AMSTAR2 tool. The included studies covered a comprehensive range of interventional approaches to modulate GM and evaluated the impact of regulating GM on T2DM. The studies were published between 2016 and 2024, and all were included in RCT clinical studies. The interventions used were mainly probiotics, dietary modifications, and herbal interventions. These studies focused on glycolipid metabolism, inflammatory markers, and GM changes. However, the quality of these studies varied, with only 1 study rated as high quality, 6 studies as low quality, and 16 studies as very low quality. Overall, these studies were relatively well evaluated for regulating GM to improve T2DM, but there are still many opportunities to improve the quality of evidence from studies in the future.

### 4.1 Overview of modulating GM to improve T2DM

Consolidation of the available evidence suggests that modulating GM has a positive effect on some of the indicators, but the results are not uniform. Thirteen studies confirmed that modulating GM significantly improved FBG (59.09%), 18 studies confirmed that modulating GM significantly improved HbA1c (81.82%), 12 studies confirmed that modulating GM significantly improved HOMA-IR (63.16%), 8 studies confirmed that modulating GM significantly improved FIns (72.73%), compared with the control group, and 7 studies confirmed that modulating GM significantly improved TC (70%), 4 studies confirmed that modulating GM significantly improved TG (70%), 5 studies confirmed that modulating GM significantly improved HDL-C (50%), 3 studies confirmed that modulating GM significantly improved LDL-C (33.33%), 4 studies confirmed that modulating GM significantly improved CRP (44.44%), 1 study confirmed that regulating GM significantly improved IL-6 (33.33%), 1 study confirmed that regulating GM significantly improved IL-12 (100%), 2 studies confirmed that regulating GM significantly improved LPS (100%), 2 studies confirmed that regulating GM significantly improved TNF-α (40%), and 4 studies confirmed that regulating GM significantly improved BMI (57.14%), and 1 study confirmed that regulating GM significantly improved Body Weight (100%).Overall, the application of regulating GM, especially probiotics/haplochemicals, showed relatively consistent positive trends in improving glycemic indicators such as HbA1c and FBG. In contrast, there was more heterogeneity in the effects on lipid and inflammatory indicators. The reason for this phenomenon may be due to differences in intervention type/strain, population characteristics, and different sensitivities of outcome indicators.

We found that there are various ways to modulate GM in the treatment of T2DM. Of these, probiotics/haplochemicals are the most commonly used interventions, followed by prebiotics/dietary fiber and dietary pattern modification. The combination of metformin and probiotics or herbs is also becoming popular. Despite the favorable results of FMT in animal experiments, its use in clinical trials does not seem to be widespread. This may be related to the ethical requirements for clinical trials.

Although clinical studies on this topic have been widely conducted, the quality of the studies is not satisfactory from the evidence we collected. Of the 23 studies, only 1 was assessed as high quality and the rest were of low or very low quality. Most studies did not complement the original studies outside the electronic database, which may have led to the omission of data. Some of the studies did not explore heterogeneity and publication bias in sufficient depth, which may have had an impact on the results.

### 4.2 Research progress in regulating GM to improve T2DM

#### 4.2.1 GM dysregulation characteristics and T2DM pathomechanisms

Patients with T2DM show characteristic alterations in GM at the phylum, genus, and species levels. In terms of bacterial composition, patients with T2DM have an elevated ratio of phylum Fusarium thick-walled/Bacteroides anomalies (F/B ratio), a change that has been associated with enhanced energy absorption and an increased risk of obesity (73). Specifically, butyric acid-producing bacteria were significantly reduced in T2DM patients, while the abundance of conditionally pathogenic bacteria was increased (74). The decrease in butyric acid-producing bacteria diminishes their protective and anti-inflammatory effects on the intestinal barrier. In contrast, the increase in Gram-negative bacteria, such as *E. coli*, leads to an increased release of endotoxin, activation of systemic chronic inflammation, and interference with insulin signaling (73). In addition to bacteria, the role of fungal and viral groups in the pathogenesis of T2DM is gradually being recognized (75, 76). Candida albicans disrupts the barrier function by tightly adhering to the intestinal epithelium, increasing intestinal permeability and allowing endotoxin to enter the circulatory system. Meanwhile, Candida albicans metabolites activate immune cells to release inflammatory factors such as tumor necrosis factor-α (TNF-α) and aggravate insulin resistance (IR) (77, 78). In terms of the virome, changes in phage composition indirectly affect microbiota homeostasis by lysing specific bacteria (79).

The intestinal barrier, which consists of intestinal epithelial cells, intercellular tight junctions, and a surface mucus layer, is an essential line of defense that prevents bacteria and toxins in the gut from entering the circulation. Intestinal barrier dysfunction is commonly observed in patients with T2DM, resulting in the phenomenon of leaky gut (80, 81). *Akkermansia muciniphila*, an important mucus layer symbiotic bacterium, was found to be significantly reduced in T2DM patients (82). It maintains barrier integrity by enhancing mucin expression and promoting the distribution of tight junction proteins (e.g., occludin, claudin-1). Decrease in its number leads to thinning of the mucus layer and impaired barrier function (83). Barrier damage allows endotoxins such as LPS to translocate into the portal circulation, triggering systemic chronic low-grade inflammation (84). LPS induces the expression of inflammatory factors (e.g., TNF-α, IL-6) through activation of the Toll-like receptor 4 (TLR4)/nuclear factor κB (NF-κB) signaling pathway. These inflammatory factors can interfere with the tyrosine phosphorylation of insulin receptor substrates (IRSs), block the insulin signaling pathway, ultimately leading to IR (85, 86).

GM participates in the regulation of host glycolipid metabolism through the production of a variety of bioactive metabolites that act as signaling molecules to influence host metabolic homeostasis. SCFAs are primarily produced by GM through anaerobic fermentation of indigestible carbohydrates and host secretions, chiefly comprising acetate, propionate, and butyrate. SCFAs regulate the G protein-coupled receptor (GPR41/43) and the inhibition of histone deacetylase enzymes (HDACs) through the activation of energy metabolism (87, 88). Among them, butyrate promotes secretion of GLP-1 from intestinal L cells, increases insulin secretion and improves pancreatic β-cell function (89). Bile acid (BAs) metabolism is another meaningful way in which microbiota influence host metabolism. Primary bile acids are de-converted to secondary bile acids in the intestine by de-conjugation by colony bile salt hydrolases. These secondary bile acids participate in the regulation of glucose homeostasis and energy expenditure as natural ligands for farnesol X receptor and G protein-coupled bile acid receptor 1 (90). Other metabolites such as trimethylamine oxide (TMAO), branched-chain amino acids (BCAAs) and imidazole propionic acid are also involved in T2DM pathogenesis (91). For example, TMAO promotes insulin resistance by activating the NLRP3 inflammatory vesicle; BCAAs interfere with glucose uptake by inhibiting phosphorylation of key proteins in the insulin signaling pathway; and the microbial metabolite imidazole propionic acid has recently been found to impair the glucose-lowering effect of metformin by inhibiting AMPK phosphorylation via p38γ kinase.

#### 4.2.2 Therapeutic strategies and clinical evidence for modulating GM

Probiotics and prebiotics. The most direct intervention strategy for regulating GM is with specific probiotic strains, which show apparent efficacy in managing T2DM. *Akkermansia muciniphila* has received much attention as a new generation of functional probiotics. In a recent study (82), it was found that supplementation with AKK-WST01 significantly increased colonization rates and improved body weight, visceral fat, and glucolipid metabolism only in patients with low baseline intestinal AKK levels, but not in patients with high baseline levels. Based on this finding, a new concept of “individualized and precise probiotic supplementation guided by the basal level of intestinal bacteria” was proposed, which provides an important paradigm for precise probiotic intervention. This finding underscores the critical importance of a precision medicine approach in modulating the gut microbiota for therapeutic purposes. The efficacy of Akkermansia muciniphila supplementation being contingent upon its pre-existing intestinal abundance perfectly illustrates a core tenet of precision medicine: stratifying patient populations based on individual biomarkers (here, baseline microbial abundance) to predict and optimize treatment response. This moves beyond the traditional “one-size-fits-all” probiotic model towards a more targeted strategy, ensuring that interventions are directed to those most likely to benefit, thereby enhancing efficacy and potentially reducing unnecessary supplementation. Implementing this precision paradigm requires the development of reliable, accessible diagnostic tools to quantify specific bacterial baselines routinely. Future research should focus on defining clinically relevant cut-off values for “low” versus “high” baseline levels and exploring the mechanisms underlying the differential response. Furthermore, this concept may extend beyond Akkermansia muciniphila to other probiotic candidates and synbiotic formulations, advocating for a fundamental shift in the design and evaluation of microbiota-targeted therapies. Ultimately, integrating microbial profiling into patient assessment could pave the way for truly personalized nutrition and microbiome-based medicine in the management of metabolic disorders like T2DM.

Traditional probiotics such as *Bifidobacterium* and *Lactobacillus* have also shown clinical value. Studies have shown that supplementation with *Bifidobacterium bifidum* reduces FBG and HbA1c by mechanisms involving reduction of endotoxemia and enhancement of intestinal barrier function (92). In contrast, *Lactobacillus* plantarum P9 improves insulin sensitivity by increasing the abundance of butyric acid-producing bacteria and decreasing the levels of inflammatory factors (93). It is worth noting that the effects of probiotics are strain-specific, and the metabolic effects of different strains or even other strains of the same bacterial species may vary significantly (94). Prebiotics, as substrates that are not digested by the host but can be utilized by beneficial bacteria, have equally important regulatory roles. Oligofructose (FOS) and inulin, among others, selectively promote the proliferation of bifidobacteria and lactobacilli (95). Synergistic preparations (combination of probiotics and prebiotics) have shown synergistic effects in improving insulin resistance and are expected to be the future direction of application (96).

FMT is a therapeutic approach to re-establish standard GM structure by transplanting functional GM from the feces of a healthy donor into the intestines of a patient (97). Several studies have confirmed that FMT partially restores intestinal GM diversity and improves metabolic indices in patients with T2DM. A non-blinded, single-arm intervention trial that included 17 patients with T2DM found significant decreases in HbA1c and fasting glucose and a significant increase in postprandial C-peptide levels in patients after FMT (41). Another study compared the effects of dietary intervention and diet combined with FMT and found more rapid metabolic improvement in the combined group (98).

Diet is one of the most effective natural means of regulating GM. Results from a large cohort study showed that about one-third of the more than 500 blood metabolites associated with impaired glycemic control were associated with GM alterations and confirmed that short-term lifestyle changes (e.g., the Mediterranean diet or exercise) specifically modulate GM-related metabolites (99). A high-fiber diet promotes the production of SCFAs and improves insulin sensitivity (100); a Mediterranean diet (rich in unsaturated fatty acids and polyphenols) increases *Bifidobacterium* and *Lactobacillus* abundance and reduces inflammatory responses (101).

There is a complex bidirectional interplay between commonly used hypoglycemic drugs and GM. On the one hand, drugs directly affect GM composition and function; on the other hand, GM characteristics determine drug metabolism and efficacy, providing the basis for individualized treatment. Metformin, as a first-line hypoglycemic agent, achieves its effect partly by regulating GM. Studies have shown that metformin increases the abundance of *Ackermannia* and some SCFAs-producing bacteria and improves metabolic function by decreasing *Bacteroides fragilis*, which mediates the glycine ursodeoxycholic acid (GUDCA)-gut FXR axis (102). However, another recent study found that the potent BSH activity of Bacteroides fragilis led to dysregulation of bile acid metabolism and impaired metformin efficacy via the FXR-ceramide axis in T2DM patients enriched with *Bacteroides vulgatus* (*Phocaeicola vulgatus*). Targeting this population, inhibition of the bacterium with cefaclor or improvement of mitochondrial function with adenosylcobalamin restores metformin efficacy and provides a new strategy for overcoming resistance (103). The cardiovascular protective effects of SGLT2 inhibitors, such as empagliflozin, have also been linked to bacterial regulation. Clinical studies have found that SGLT2i reduces *Clostridium difficile* (Fusobacterium) abundance and decreases the risk of cardiovascular events in patients with T2DM, which may be associated with decreased levels of inflammatory factors and improved endothelial function (104).

Metabolic surgery is an effective treatment for obesity with T2DM and its mechanism of action is also closely related to GM remodeling. Significantly enhanced intestinal glucose excretion was observed in T2DM patients undergoing Roux-en-Y gastric bypass (RYGB) and was negatively correlated with postoperative glucose levels. Animal models further validated that surgery-induced changes in GM are a direct causal factor in promoting intestinal glucose excretion and improving glucose metabolism. Biliopancreatic Limb (BPL) length was also found to influence intestinal glucose excretion after RYGB, with the long BPL group exhibiting better glycemic control (105).

**Figure 5:**
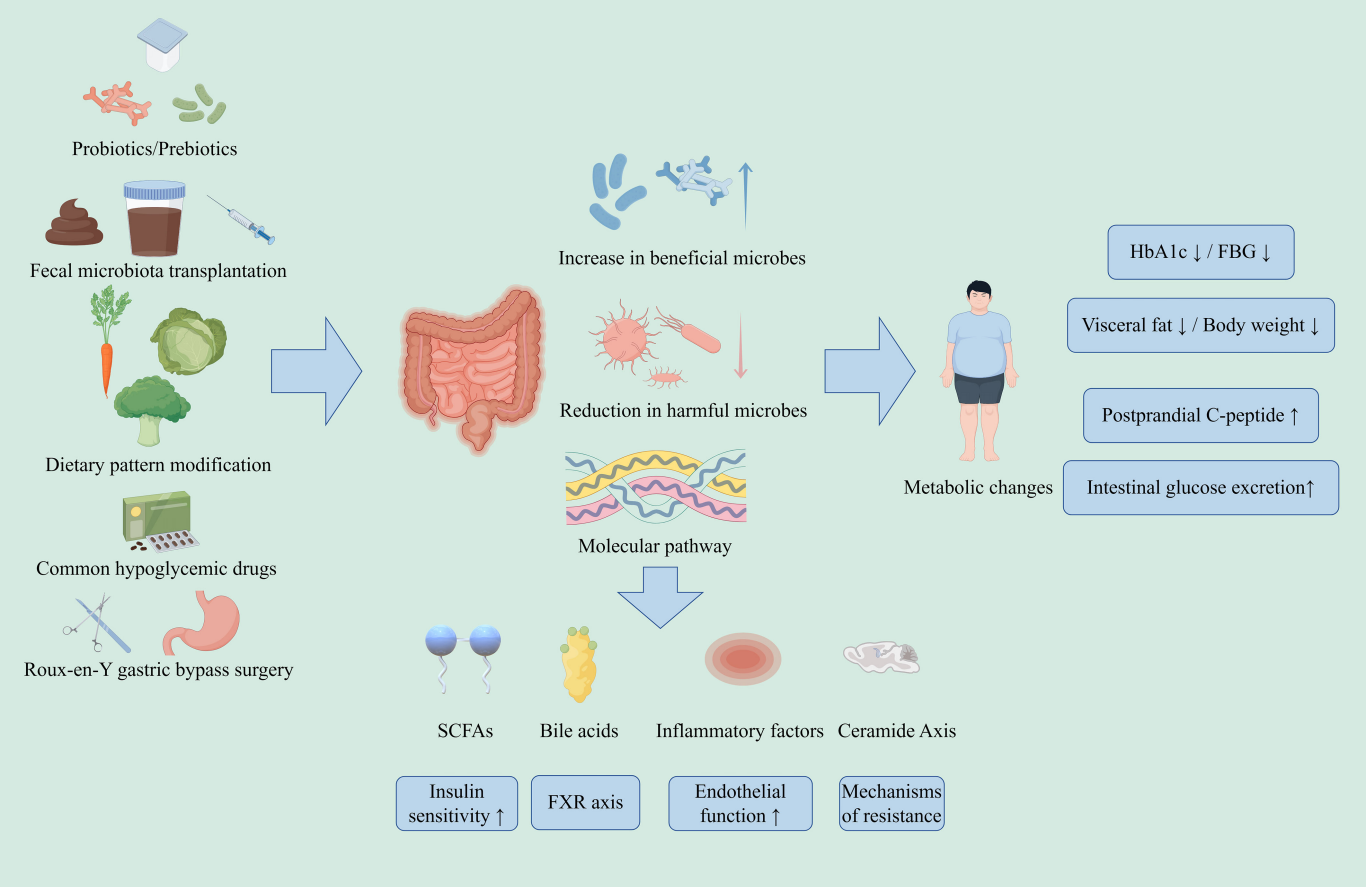
Key clinical strategies and routes of action for regulating GM to improve T2DM.

As a key environmental factor in the pathogenesis of T2DM, GM is involved in various pathological processes such as IR, β-cell dysfunction and chronic inflammation through the “GM-metabolite-host” axis. **Figure 5** shows the current major clinical strategies and routes of action for regulating GM to improve T2DM. Important advances in recent years can be summarized in three points: first, the central role of GM metabolites in signal transduction has been revealed; second, it has been clarified that part of the effects of traditional therapeutic agents (e.g., metformin, SGLT2i, and RYGB) are achieved through colony modulation; and third, it has been demonstrated that interventional strategies to regulate GM are of clinical value in the management of T2DM. The current research trend is shifting from describing GM correlations to causal mechanism resolution and precise intervention exploration. Future research needs to address key issues such as individual heterogeneity, establishment of multi-omics predictive modeling, and development of engineered microbiota therapy.

## 5 Strengths and limitations

This study consolidated the existing evidence, combed through the current status of regulating GM to improve T2DM, and identified gaps in current research. It also contains some limitations that should be considered. First, we included systematic reviews and meta-analyses as part of the retrieval process, which resulted in previously published studies that may have been overlooked. Second, most of the search results were systematic reviews and meta-analyses written in English, which may have missed studies in other languages. Third, although the effects of multiple interventions were evaluated, this result may not be satisfactory because the included studies differed in terms of subjects, interventions, controls, and outcomes. Fourth, the included studies all lacked follow-up information to provide more details about long-term outcomes and patients’ quality of life.

## 6 Conclusion

This scoping review integrates current evidence from systematic evaluations and Meta-analyses of modulating GM to improve T2DM. The results showed that probiotics/coagulants were the dominant intervention modality, with most studies reporting improvements in some metabolic markers such as glucose and lipids, but with heterogeneity of results and generally low methodological quality (only 1 high-quality). Particularly noteworthy is the serious lack of assessment of intervention safety in the included studies. Although the available evidence suggests potential, limited by study quality, heterogeneity, and lack of safety data, its clinical translational value needs to be confirmed by more rigorous randomized controlled trials with large samples, long-term follow-up, standardized reporting of adverse events, and their high-quality systematic evaluations.

## Data Availability

The original contributions presented in the study are included in the article/**Supplementary Material**. Further inquiries can be directed to the corresponding author.
