# Supplementary material for: Targeting the gut microbiome for type 2 diabetes management: a scoping review of systematic reviews and meta-analyses
Source: Front Endocrinol (Lausanne). 2026 Jan 29;17:1682174. doi: 10.3389/fendo.2026.1682174 (PMC12894762; doi:10.3389/fendo.2026.1682174)
Supplement: Supplementary file 1 [file Table1.docx]

Table 2 Detailed information on SR/ME included

| First author | Year | Journal | Funding | Diagnostic standards | Age | Special population | Intervention | Aims | RCTs only | Databases searched | Number of researches / sample size | Outcomes | Main results |
| --- | --- | --- | --- | --- | --- | --- | --- | --- | --- | --- | --- | --- | --- |
| Caifeng Li | 2016 | Medicine | No | NR | None | None | The intervention was probiotic agent, and placebo was applied as comparison to the intervention. | The aim of the study is to assess the effect of probiotic supplementation on metabolic profiles in T2DM. | Yes | PubMed, EMBASE, Cochrane Library. | 12(714) | 1.FBG. 2.HDL-C 3.LDL-C. 4.TC. 5.TG. 6.HbA1c. 7.HOMA-IR. | In conclusion, this meta-analysis of available RCTs suggests that probiotics has beneficial effect on FBG and HDL-C in T2DM. Probiotic agents might become a new method for management of T2DM. However, before they can be recommended for use in supportive treatment of T2DM, large scale and long duration multicenter randomized controlled trials are still required. |
| Colantonio, Angela G. | 2020 | Journal of The Academy of Nutrition And Dietetics | No | NR | ≥18 | None | The intervention had to include the use ofa prebiotic or substance with prebiotic properties. | The objective of this systematic review was to evaluate the effect of prebiotics and substances with prebiotic properties on the metabolic and inflammatory biomarkers of individuals with T2DM compared with placebo. | Yes | PubMed,  Science Direct, Cochrane Central Register of Controlled Trials. | 27(1072) | 1.HbA1c 2.FBG 3.SBP  4.DBP 5.TC 6.LDL-C 7.HDL-C 8.TG 9. Body Weight 10.BMI 11.CRP  12.IL-6 13.IL-12 14.LPS 15.TNF-α 16.Catalase  17.GSH-Px 18.SOD 19.TAC 20.IL-4 | There is fair evidence that prebiotics and substances with prebiotic properties may improve metabolic and inflammatory biomarkers related to T2DM in women who are at least age 18 years. |
| Edris Ardeshirlarijani | 2019 | Daru : Journal of Faculty of Pharmacy, Tehran University of Medical Sciences | Tehran University of Medical Sciences & health services grant numbered 39856–192–03-97 | NR | None | None | Test group:probiotics or prebiotics Control group:a placebo or other treatments. | The role of oxidative stress in pathogenesis of diabetes is well established. | Yes | PubMed,  Web of Science, Scopus | 13(840) | 1.HbA1c 2.TAS 3.MDA 4.GSH 5.NO 6.FBG | In conclusion, our results revealed a beneficial effect of probiotics on reducing FBS, or oxidative stress biomarkers in T2DM patients without any significant improvement of glycemic status. Hence, probiotics might be considered as potential target for care and management of T2DM. However, larger well-designed and long duration RCTs are required before making a conclusive recommendation for subjects with T2DM. In addition, due to potential safety concerns in at-risk individuals such as elderly careful selection criteria need to be considered in designing RCTs. |
| Guang Li | 2023 | Journal of Translational Medicine | National Natural Science Foundation of China (No. 81400857), Natural Science Foundation of Guangdong Province (No.2021A1515011527), Pearl River S&T Nova Program of Guangzhou (No. 201610010093). | NR | ≥18 | None | Test group:the intake of probiotics from supplements and/or food. Control group: received a placebo intervention | This systematic review and meta-analysis study aimed to evaluate the effectiveness of probiotics supple‑ mentation on glycaemic control in patients with type 2 diabetes mellitus (T2DM) based on the data from the ran‑ domised clinical trials (RCTs). | Yes | PubMed,  Web of Sciences, Embase,  Cochrane Library | 30(1827) | 1.FBG 2.FIns 3.HbA1c 4.HOMA‑IR | This systematic review and meta-analysis summarised data from 30 RCTs, including a total of 1,827 individuals, to evaluate the effects of probiotic supplementation on glycaemic control in T2DM patients. The results revealed that probiotic supplementation significantly decreased FBG, insulin, and HbA1c levels and HOMA-IR scores in T2DM patients. Further subgroup analyses showed that the effect was larger in the subgroups of Caucasians, high baseline BMI , Bifidobacterium probiotics, and food-type probiotics. |
| Hamda Memon | 2023 | Diabetes Research and Clinical Practice | Khalifa University research grant. | NR | Adult | None | Test group:metformin plus probiotics. Control group: metformin. | This systematic review and meta-analysis aim to assess the efficacy of metformin plus probiotics versus metformin alone on outcomes in patients with T2DM. | Yes | MEDLINE,  EMBASE | 14(1009) | 1.FBG 2.HbA1c 3.FIns 4. HOMA-IR 5. HDL-C 6.LDL-C 7.TC 8. Gastrointestinal intolerance | The addition of probiotics to metformin therapy is associated with improvement in T2DM outcomes. |
| Hao Zhong | 2024 | Biology | China Postdoctoral Science Foundation (No. 2022M721715), and Key Laboratory of Tropical Fruits and Vegetables Quality and Safety for State Market Regulation (KF-2023014). | NR | None | None | Test group: Probiotic fermented milk supplementation. Control group: Conventional fermented milk or non-dairy controls/placebos. | The present meta-analysis was performed to assess the effects of probiotic fermented milk supplementation on glucose and lipid metabolism parameters and inflammatory markers in patients with T2DM using published data from randomized controlled trials (RCTs). | Yes | PubMed,  Web of Science,  Cochrane Library | 10(525) | 1.FBG 2.HOMA-IR 3.HbA1c 4.TC 5.LDL-C 6.HDL-C 7.CRP 8.TNF-α | The present meta-analysis was performed to assess the effects of probiotic fermented milk supplementation on glucose and lipid metabolism parameters and inflammatory markers in patients with T2DM using published data from randomized controlled trials (RCTs). |
| Ismat E Ayesha | 2023 | Cureus Journal of Medical Science | No | NR | None | None | Test group: probiotics. Control group: placebo. | This article aims to review the clinical evidence present in the form of RCTs on the clinical efficacy of probiotics’ use in T2DM patients while comparing its short-term effect (taken as 8 weeks or less) versus the long-term (taken as 12 weeks or more). | Yes | Medline,  PubMed （2017-2023） | 22(2218) | 1.HOMA-IR 2.FBG 3.HbA1c | This systematic review indicates that probiotics have the potential to lower baseline levels of HbA1c, FBG, and HOMA-IR in T2DM patients. Furthermore, the study highlights the significant contribution of gut microbiota to the development of T2DM, with dysbiosis potentially linked to obesity and insulin resistance. |
| Kecheng Yao | 2017 | Medical Science Monitor : International Medical Journal of Experimental And Clinical Research | Graduate Student Research Innovation Fund of Three Gorges University (SDYC2016090);  Science and Technology Research and Development Project of Yichang City (A11301-15) | NR | None | None | Test group: probiotics as a dietary supplementation. | This meta-analysis aimed to summarize the effect of probiotics on glucose and lipid metabolism and C-reactive protein (CRP) from 12 randomized controlled trials (RCTs). | Yes | PubMed,  MEDLINE,  EMBASE,  Cochrane library | 12(684) | 1.FPG 2.HbA1c 3.FIns 4. HOMA-IR 5.CRP 6.TC 7.LDL-C 8.HDL-C | This meta-analysis demonstrated that probiotics supplementation was associated with significant improve- ment in HbA1c and fasting insulin in type 2 diabetes patients. More randomized placebo-controlled trials with large sample sizes are warranted to confirm our conclusions. |
| Lina Ding | 2021 | Frontiers in Pharmacology | Key Research and Development Program of Shandong Province (2018YYSP030), Natural Science Foundation of Shandong Province (ZR2017YL009, ZR2019PH096), Innovation Project of Shandong Academy of Medical Sciences, and the Shandong Province “Double-Hundred Talent Plan” on 100 Foreign Experts and 100 Foreign Expert Teams Introduction (WST2018004). | NR | ≥18 | None | Test group:probiotic supplementation. | This study aims to investigate the comprehensive effects of probiotics on inflammatory markers in adults with T2DM. | Yes | PubMed,  Embase,  Cochrane Library,  Web of Science | 17(836) | 1.TNF-α 2.IL-6 3.CRP 4.FPG 5.HbA1c 6.HOMA-IR | The evidence supports that probiotic supplementation had beneficial effects on some inflammatory markers (TNF-α and CRP) in parallel with improving glucose homeostasis (FPG, HbA1c, and HOMA-IR) in adults with T2DM. Probiotic supplementation could be beneficial for T2DM patients due to the attenuation of chronic low-grade inflammation by the gut microbiota, which was modulated by probiotics. |
| Marta A Kasińska | 2015 | Polskie Archiwum Medycyny Wewnetrznej | Grant (no. 503/0-077-09/503-01-002; to the De- pertment of Internal Diseases, Diabetology and Clinical Pharmacology ) from the Medical Univer- sity of Lodz, Poland, and partially sponsored by the Polish Society of Metabolic Diseases. | NR | None | None | Test group:probiotics. Control group:placebo. | The aim of the study was to assess the ability of probiotics to modify selected cardiometabolic risk factors in subjects with type 2 diabetes. | Yes | PubMed,  Embase,  Cochrane Library,  Scopus | 8(438) | 1.FPG 2.HbA1c 3.HOMA-IR 4.FIns 5.HDL-C 6.CRP | The meta-analysis showed a significant effect of probiotics on reducing HbA1c levels and HOMA-IR. Supplementation with probiotics did not have a significant effect on FPG, insulin, and CRP levels as well as the lipid profile. |
| Omorogieva Ojo | 2020 | Nutrients | No | NR | Adult | None | Dietary fibre including a macrobiotic diet. | To evaluate the role of dietary fibre in modulating gut microbiota dysbiosis in patients with type 2 diabetes. | Yes | EBSCOHost | 9(713) | 1.Relative abundance of gut microbiota (genera only), 2. SCFAs, 3.FBG, 4.HbA1c 5.HOMA-IR, 6.Adverse events. | Dietary fiber significantly improved the relative abundance of bifidobacteria, total SCFA, and glycosylated hemoglobin. However, dietary fiber did not appear to have a significant effect on fasting glucose, HOMA-IR, acetic acid, propionic acid, butyric acid, and adverse events . |
| Omorogieva Ojo | 2021 | Nutrients | No | NR | None | None | The provision of almonds or advice to increase almond consumption. | To evaluate the effects of almonds on gut microbiota, glycometabolism, and inflammatory parameters in patients with type 2 diabetes. | Yes | EBSCO-host, EMBASE,  Google Scholar | 8(221) | 1.Gut microbiota, 2.HbA1c, 3.BMI, 4.FBG, 5.2hPG, 6. HOMA-IR, 7.GLP-1, 8. FIns  9.CRP  10.TNF-α | An almond-based diet may be effective in promoting short-chain fatty acid-producing bacteria and reducing glycated hemoglobin and body mass index in patients with T2DM. However, the effects of almonds on fasting glucose, 2-hour postprandial glucose, inflammatory markers (C-reactive protein and TNF-α), GLP-1, HOMA-IR, and fasting insulin were not significant . |
| Omorogieva Ojo | 2021 | Nutrients | No | NR | ≥18 | None | Dietary fibre including microbiotic diet was the intervention. | The aim of this review is to examine the effect of dietary fibre on gut microbiota, lipid profile, and inflammatory markers in patients with type 2 diabetes. | Yes | EBSCO-host, EMBASE,  Google Scholar, Reference lists | 10 | 1.Gut microbiota, 2.LPS, 3.LBP, 4.HDL-C, 5.LDL-C, 6.TC, 7.TG, 8.CRP, 9.IL-6, 10.TNF-α, 11.Adiponectin, 12.Leptin, 13.BMI | The findings of this review have shown that dietary fibre can significantly (p < 0.05) increase the relative abundance of Bifidobacterium and significantly decrease (p < 0.05) lipopolysaccharide, total cholesterol, and body mass index as compared with a control. However, the results demonstrated that there were no significant (p > 0.05) differences between the dietary fibre group and a control with respect to LBP, triglyceride, HDL cholesterol, LDL cholesterol, IL-6, TNF-α, adiponectin, and leptin. These findings have public health implications in terms of the use of dietary fibre in nutritional interventions and as strategies for managing type 2 diabetes. |
| Rui Xiao | 2023 | Nutrients | National Natural Science Foundation of China (No. 31972052, 32021005, 31820103010), the Fundamental Research Funds for the Central Universities (JUSRP22006, JUSRP51501), the Program of Collaborative Innovation Centre of Food Safety and Quality Control in Jiangsu Province, Wuxi key medical discipline construction program fromWuxi Health Commission—Innovation Team of Anaesthesiology (CXTD2021017, 2021–2025) | NR | ≥18 | No allergy to probiotic components. | Test group: probiotics. Control group: placebo. | Our aim was to identify the characteristics of potentially practical interventions—such as the number of strains used, the route of administration, the length of a course of therapy, and the probiotics’ ability to adapt to different physiological states of a host—and to assess the general clinical efficacy of probiotic supplementation for the treatment of T2DM. | Yes | PubMed,  Embase,  Cochrane Library,  Web of Science | 37(2502) | 1.HbA1c. 2.FIns. 3.FBG. 4.HOMA-IR. 5.TC. 6.TG. 7.LDL-C. 8.HDL-C. 9.BMI | In conclusion, this meta-analysis shows that oral probiotics aid in the regulation of glucolipid metabolism in patients with T2DM, primarily indicated by a marked decrease in glucose metabolism and lipid metabolism following treatment. These findings suggest that probiotic supplementation can be utilised as a complementary therapy to help prevent T2DM. To confirm the ability of probiotics to support glycaemic, lipid, and blood pressure regulation, additional clinical investigations with various patient profiles, probiotic dosages, and intervention durations are required. |
| Ting Mao | 2021 | Journal of Functional Foods | No | NR | None | None | Increase in dietary fiber intake, either from diet or fiber supplement, for glycemic control. | The aims of this study are as follows: 1) to conduct a comprehensive meta-analysis to investigate the extent to which dietary fiber, either from diet or supplements, can improve glycemic and insu­ linemic control – HbA1c, fasting blood glucose (FBG), fasting insulin, Homeostatic Model Assessment for Insulin Resistance (HOMA-IR), 2-h postprandial glucose, and 2-h postprandial insulin – in patients with T2DM, 2) to detect the potential relationship between dietary fiber and BMI, and 3) to explore the tolerable amount of dietary fiber for these patients. | Yes | PubMed (MEDLINE), Embase,  Ovid,  Web of Science, Cochrane Library | 22(911) | 1.HbA1c 2.FBG 3.FIns 4.HOMA-IR 5.2hPG 6.2hPI 7.FBG 8.BMI | In conclusion, both soluble fiber products and fiber from natural foods are effective in improving glycemic control and insulin sensitivity in T2DM patients, the former yielding better effects. However, due to most of the evidence from rather short-term trials and the detected significant between-study heterogeneity, further long-term and high-quality RCTs are needed. |
| Vajihe Akbari | 2016 | Nutrition in Clinical Care | Isfahan University of Medical Sciences (grant no. 295082). | NR | ≥18 | None | Test group:Probiotic supplementation Control group:Placebo (product without microorganisms) administration | The aim of this systematic review was to assess the effects of probiotic consumption on glycemic control in diabetic patients. | Yes | PubMed,  Scopus,  ISI Web of Knowledge,  Cochrane Library, ProQuest Dissertations, ClinicalTrials.gov | 13(455) | 1.FBG 2.FIns 3.HOMA-IR 4.HbA1c 5.Adverse events | Administration of probiotics appears to have a beneficial role in the management of type 2 diabetes; however, more clinical studies with adequate sample sizes and sound methodology are required to inform the development ofevidence-based treatment guidelines. |
| Xiaoyu Xu | 2024 | Frontiers in Nutrition | Humanities and Social Sciences Planning Fund Project of the Ministry of Education ofChina (No. 15YJAZH085). | NR | None | None | Specific dietary patterns or combinations of diets. | To assess the impact of dietary interventions on improving gut microbiota and metabolic levels in patients with type 2 diabetes. | Yes | PubMed,  EMBASE,  Web of Science, ScienceDirect. | 12(676) | 1.Gut microbiota diversity and richness, 2. composition of the gut microbiota, 3.Gut microbiota function, 4.FBG, 5.HbA1c, 6.TC, 7.LDL-C, | The high dietary fiber regimen resulted in significant reductions in FBG, HbA1c, and total cholesterol levels, while increasing the prevalence of short-chain fatty acid-producing bacteria. High-fat and low-carbohydrate diets were particularly effective in reducing HbA1c levels, and low-fat, low-carbohydrate diets demonstrated significant reductions in FBG, HOMA-IR, BMI, and waist circumference. |
| Xinghui Wang | 2024 | Frontiers in Endocrinology | National Natural Science Foundation of China (No.81960805),  Guizhou Provincial Health Commission (No.gzwkj2024-062),  Guizhou Provincial Health Commission (No.gzwkj2024-255). | NR | ≥18 | None | Test group: probiotics. Control group: conventional treatment or placebo. | To comprehensively evaluate how various probiotic intervention times affect glycemic control in people with T2DM. | Yes | PubMed, Embase, Web of Science, Cochrane Library | 8(507) | 1.HbA1c 2.FIns 3. HOMA-IR 4.FBG 5.BMI | This meta-analysis found probiotics at different intervention times play a positive role in modulating glucose in T2DM, specifically for HbA1c in six to eight weeks, Insulin and HOMA-IR in six to eight weeks, and 12-24 weeks. To confirm our findings, further excellent large-sample research is still required. |
| Yan Yang | 2024 | Endocrine | Handan Science and Technology Research and Development Plan (No.19422083008),  Biomedical Joint Fund of Natural Science Foundation of Hebei Province (No. H2021402005), Project Fund of Clinical Medicine Excellent Talents funded by Hebei Provincial Department of Finance (No.: [2020] No.23). | NR | 20–80 years | None | FMT | We conducted a meta-analysis and systematic review of existing randomized controlled trials (RCTs) to assess the efficacy of FMT in T2DM. | Yes | PubMed, Embase, Cochrane Library,  Web of Science | 4(140) | 1.HbA1c 2.FPG 3.2hPG 4.HOMA-IR 5.TG 6.TC 7.LDL-C 8.HDL-C 9.BMI 10.AST 11.ALT 12.SBP 13.DBP 14.Changes in intestinal flora | Notably, the combination of FMT with other treatments exhibited a more pronounced effect. Following both single FMT and combined FMT treatment, patients with type 2 diabetes exhibited reductions in levels of triglycerides, postprandial blood glucose, homeostatic model assessment of insulin resistance, total cholesterol, alanine aminotransferase, and diastolic blood pressure.Moreover, our investigation revealed the ability of FMT to reshape the intestinal microbiota, restoring the equilibrium of dominant microbial communities. |
| Yimeng Hu | 2017 | Medicina Clinica | National Natural Science Foundation of China (Grant No. 81370872). | NR | None | None | Test group:consumption of probiotics or synbiotics. Control group:placebo | To objectively evaluate the effects of probiotics supplement on glycemic control and lipid metabolism in patients with type 2 diabetes mellitus (T2DM). | Yes | PubMed, Cochrane Central Register of controlled trials, Embase, ISI of knowledge databases, Scopus | 12(770) | 1.FBG 2.FIns 3.HbA1c 4.HOMA-IR 5.QUICKI 6.TC 7.TG 8.HDL-C 9.LDL-C | We analyze the beneficial effects of probiotics, especially Lactobacillus and Bifidobacterium on managing diabetes in this paper. As a potential biotherapeutics, probiotics can improve glucose control and lipid metabolism under the conditions of full dose and long treatment course. |
| Yujiao Zheng | 2020 | Phytomedicine | National Natural Science Foundation of China (No. 81973837) | NR | None | None | Chinese herbal compounds, Chinese patent medicine, and  single Chinese medical herbs, which can be administered in the form of  decoctions, granules, or powders. | To systematically review all randomized controlled trials (RCTs) on TCM for gut microbiota to assess the effectiveness and safety of TCM in T2DM patients. | Yes | Web of Science, PubMed,  EMBASE, Cochrane Library, China National Knowledge Infrastructure,  Wanfang Database,  IP Information Chinese Scientific Journal Database | 5(650) | 1. Modulation of gut microbiota; 2. HbA1c 3.FPG 4.2hPG 5.HOMA-IR 6.HOMA-β | TCM has the ability to regulate intestinal flora and improve glucose metabolism in T2DM patients. |
| Yunwen Tao | 2020 | Journal of Translational Medicine | National Natural Science Foundation of China (31771417, 31571291) ,  Priority Academic Program Development (PAPD) of Jiangsu higher educa‑ tion institutions. | NR | None | None | Test group: probiotic, Control group: placebo was applied as comparison to the intervention. | The purpose of the present study was to evaluate the effectiveness of probiotics on type II diabetes mel‑ litus (T2DM). | Yes | PubMed,  Web of Science,  China National Knowledge Infrastructure,  Chinese Scientific Journal Databases,  Wan Fang database, China biology medicine disc | 15(902) | 1.HbA1c 2.FBG 3.HOMA-IR | The results of our meta-analysis indicated that probiotics treatment may reduce HbA1c, FBG and insulin resistance level in T2DM patients. More clinical data and research into the mechanism of probiotics are needed to clarify the role of probiotics in T2DM. |
| Yunxi Xu | 2022 | Frontiers in Endocrinology | Sichuan Provincial Administration of Traditional Chinese Medicine (Grant Number: 2021MS457). | NR | None | None | Used treatment with CHFs and metformin. | To assess and analyse the effectiveness and safety of combined Chinese herbal formula (CHF) and metformin treatment in the modulation of the gut microbiota in the amelioration of type 2 diabetes mellitus(T2DM). | Yes | PubMed,  Web of Science, Cochrane Library, China National Knowledge Infrastructure,  Wan Fang China, VIP Medical Information,  Chinese Biomedical Literature | 12(1307) | 1.Bifidobacterium 2.Lactobacillus 3.Bacteroidetes 4.Enterobacteriaceae 5.Enterococcus 6.Saccharomycetes 7.FPG 8.2hPG 9.HbA1c 10.FIns 11.HOMA-IR | This study provides evidence to support the potential efficacy and safety of combination therapy with herbal formulas and metformin in controlling hyperglycemia and regulating intestinal flora. |
