# Supplementary material for: Targeting the gut microbiome for type 2 diabetes management: a scoping review of systematic reviews and meta-analyses
Source: Front Endocrinol (Lausanne). 2026 Jan 29;17:1682174. doi: 10.3389/fendo.2026.1682174 (PMC12894762; doi:10.3389/fendo.2026.1682174)
Supplement: Supplementary file 2 [file Table2.docx]

Table 5 Quality evaluation of included SR/ME

| First Author | Item 1 | Item 2 | Item 3 | Item 4 | Item 5 | Item 6 | Item 7 | Item 8 | Item 9 | Item 10 | Item 11 | Item 12 | Item 13 | Item 14 | Item 15 | Item 16 | Confidence Level |
| --- | --- | --- | --- | --- | --- | --- | --- | --- | --- | --- | --- | --- | --- | --- | --- | --- | --- |
| Caifeng Li 2016 | Yes | Yes | No | Partly | Yes | Yes | No | Yes | Yes | No | Yes | Yes | Yes | No | Yes | Yes | Low |
| Colantonio, Angela G. 2020 | Yes | No | Yes | Partly | Yes | Yes | No | Yes | Yes | No | Not applicable | Not applicable | Not applicable | Not applicable | Not applicable | Yes | Extremely low |
| Edris Ardeshirlarijani 2019 | Yes | Yes | No | Partly | Yes | Yes | No | Yes | Yes | No | Yes | Yes | Yes | No | Yes | Yes | Low |
| Guang Li 2023 | Yes | Yes | No | Partly | Yes | Yes | No | Yes | Yes | No | Yes | Yes | Yes | Yes | Yes | Yes | Low |
| Hamda Memon 2023 | Yes | Yes | No | Partly | Yes | Yes | No | Yes | Yes | No | Yes | Yes | Yes | Yes | No | Yes | Extremely low |
| Hao Zhong 2024 | Yes | Yes | No | Partly | Yes | Yes | No | Yes | Yes | No | Yes | Yes | Yes | Yes | No | Yes | Extremely low |
| Ismat E Ayesha 2023 | Yes | Yes | No | Partly | Yes | Yes | No | Yes | Yes | No | Yes | Yes | Yes | Yes | No | Yes | Extremely low |
| Kecheng Yao 2017 | Yes | No | No | Partly | Yes | Yes | No | Yes | Yes | No | Yes | Yes | Yes | No | No | Yes | Extremely low |
| Lina Ding 2021 | Yes | Yes | No | Partly | Yes | Yes | No | Yes | Yes | No | Yes | Yes | Yes | Yes | Yes | Yes | Low |
| Marta A Kasińska 2015 | Yes | No | No | Partly | Yes | Yes | No | Yes | Yes | No | Yes | Yes | Yes | No | No | Yes | Extremely low |
| Omorogieva Ojo 2020 | Yes | No | No | Partly | Yes | Yes | No | Yes | Yes | No | Yes | Yes | Yes | No | No | Yes | Extremely low |
| Omorogieva Ojo 2021-A | Yes | No | No | Partly | Yes | Yes | No | Yes | Yes | No | Yes | Yes | Yes | Yes | No | Yes | Extremely low |
| Omorogieva Ojo 2021-B | Yes | No | No | Partly | Yes | Yes | No | Yes | Yes | No | Yes | Yes | Yes | No | No | Yes | Extremely low |
| Rui Xiao 2023 | Yes | Yes | Yes | Partly | Yes | Yes | No | Yes | Yes | No | Yes | Yes | Yes | No | Yes | Yes | Low |
| Ting Mao 2021 | Yes | No | Yes | Partly | Yes | Yes | No | Yes | Yes | No | Yes | Yes | Yes | Yes | Yes | Yes | Extremely low |
| Vajihe Akbari 2016 | Yes | Yes | Yes | Yes | Yes | Yes | Partly | Yes | Yes | No | Yes | Yes | Yes | Yes | Yes | Yes | High quality |
| Xiaoyu Xu 2024 | Yes | No | No | Partly | Yes | Yes | No | Yes | Yes | No | Yes | Yes | Yes | No | No | Yes | Extremely low |
| Xinghui Wang 2024 | Yes | Yes | Yes | Partly | Yes | Yes | No | Yes | Yes | No | Yes | Yes | Yes | Yes | No | Yes | Extremely low |
| Yan Yang 2024 | Yes | No | No | Partly | Yes | Yes | No | Yes | Yes | No | Yes | Yes | Yes | No | Yes | Yes | Extremely low |
| Yimeng Hu 2017 | Yes | No | Yes | Partly | Yes | Yes | No | Yes | Yes | No | Yes | Yes | Yes | Yes | Yes | Yes | Extremely low |
| Yujiao Zheng 2020 | Yes | Yes | No | Partly | Yes | Yes | No | Yes | Yes | No | Yes | Yes | Yes | Yes | No | Yes | Extremely low |
| Yunwen Tao 2020 | Yes | No | No | Partly | Yes | Yes | No | Yes | Yes | No | Yes | Yes | Yes | Yes | Yes | Yes | Extremely low |
| Yunxi Xu 2022 | Yes | Yes | No | Partly | Yes | Yes | No | Yes | Yes | No | Yes | Yes | Yes | Yes | Yes | Yes | Low |

Item 1: *Did the research questions and inclusion criteria for the review include the components of PICO?*

Item 2: *Did the report of the review contain an explicit statement that the review methods were established prior to the conduct of the review and did the report justify any significantdeviations from the protocol?*

Item 3 *Did the review authors explain their selection of the study designs for inclusion in the review?*

Item 4: *Did the review authors use a comprehensive literature search strategy?*

Item 5: *Did the review authors perform study selection in duplicate?*

Item 6: *Did the review authors perform data extraction in duplicate?*

Item 7: *Did the review authors provide a list of excluded studies and justify the exclusions?*

Item 8: *Did the review authors describe the included studies in adequate detail?*

Item 9: *Did the review authors use a satisfactory technique for assessing the risk of bias (RoB) in individual studies that were included in the review?*

Item 10: *Did the review authors report on the sources of funding for the studies included in the review?*

Item 11: *If meta-analysis was performed, did the review authors use appropriate methods for statistical combination of results?*

Item 12: *If meta-analysis was performed, did the review authors assess the potential impact of RoB in individual studies on the results of the meta-analysis or other evidence synthesis?*

Item 13: *Did the review authors account for RoB in primary studies when interpreting/discussing the results of the review?*

Item 14: *Did the review authors provide a satisfactory explanation for, and discussion of, any heterogeneity observed in the results of the review?*

Item 15: *If they performed quantitative synthesis did the review authors carry out an adequate investigation of publication bias (small study bias) and discuss its likely impact on the results of the review?*

Item 16: *Did the review authors report any potential sources of conflict of interest, including any funding they received for conducting the review?*
